# The effects of temperature and pH on the reproductive ecology of sand dollars and sea urchins: Impacts on sperm swimming and fertilization

**DOI:** 10.1371/journal.pone.0276134

**Published:** 2022-12-01

**Authors:** Sara Grace Leuchtenberger, Maris Daleo, Peter Gullickson, Andi Delgado, Carly Lo, Michael T. Nishizaki

**Affiliations:** 1 Biology Department, Carleton College, Northfield, MN, United States of America; 2 Friday Harbor Laboratories, Friday Harbor, WA, United States of America; Laboratoire Arago, FRANCE

## Abstract

In an era of climate change, impacts on the marine environment include warming and ocean acidification. These effects can be amplified in shallow coastal regions where conditions often fluctuate widely. This type of environmental variation is potentially important for many nearshore species that are broadcast spawners, releasing eggs and sperm into the water column for fertilization. We conducted two experiments to investigate: 1) the impact of water temperature on sperm swimming characteristics and fertilization rate in sand dollars (*Dendraster excentricus*; temperatures 8-38°C) and sea urchins (*Mesocentrotus franciscanus*; temperatures 8-28°C) and; 2) the combined effects of multiple stressors (water temperature and pH) on these traits in sand dollars. We quantify thermal performance curves showing that sand dollar fertilization rates, sperm swimming velocities, and sperm motility display remarkably wide thermal breadths relative to red urchins, perhaps reflecting the wider range of water temperatures experienced by sand dollars at our field sites. For sand dollars, both temperature (8, 16, 24°C) and pH (7.1, 7.5, 7.9) affected fertilization but only temperature influenced sperm swimming velocity and motility. Although sperm velocities and fertilization were positively correlated, our fertilization kinetics model dramatically overestimated measured rates and this discrepancy was most pronounced under extreme temperature and pH conditions. Our results suggest that environmental stressors like temperature and pH likely impair aspects of the reproductive process beyond simple sperm swimming behavior.

## Introduction

Increases in atmospheric CO_2_ have led to dramatic warming and increased acidification of the global ocean [1, 2]. These effects are further amplified in coastal regions, where shallow waters, persistent upwelling, and substantial biological activity contribute to significant variability in temperature [3–5] and pH [6, 7]. Such changes in the physical environment can lead to a wide array of biological consequences for organisms that inhabit nearshore ecosystems. For instance, thermal variation can lead to changes in distribution [8–10], feeding rate [11, 12], respiration rate [13–15], heart rate [16, 17], symbiont loss [18–20], larval developmental rate [21–23], post-settlement growth [24–26], developmental stability [27, 28], epigenetic modification and gene expression [29–34]. Similarly, acidification can have significant effects on rates of calcification [35, 36], production of non-calcified strucutures like byssal threads [37–39], patterns of gene expression [40, 41], and larval development and behavior [22, 42, 43]. In coastal ecosystems where physical factors can vary independently, understanding the effects of multiple environmental stressors is also important [44–46]. Specifically, the combined effects of temperature and pH can have varied effects on feeding [47, 48], physiological performance [28, 49], and growth [50, 51] yielding responses that may be linear, hyperbolic, or unimodal in nature. Understanding the shape of these physiological response curves is crucial in predicting how marine organisms may or may not respond to environmental change. Thus, an investigation into the effects of multiple environmental stressors on early life-history processes associated with fertilization in marine invertebrates appears warranted.

Broadcasting spawning is a mode of reproduction adopted by many marine organisms including echinoderms such as sea urchins and sand dollars. For broadcast spawners, whose gametes are directly exposed to conditions in ambient seawater, early developmental stages are often more susceptible to environmental stress and may, therefore, represent important bottlenecks for population growth and persistence under climate change [52–54]. The degree to which future warming and acidification may or may not affect organisms depends on the shape of their thermal performance curves (TPCs; e.g., physiological optima, thermal breadth). In the northeast Pacific, echinoderms show a wide range of fertilization rates and sperm swimming velocities in response to changes in temperature and pH (Table 1) [55, 56] and for some species, faster swimming sperm may indeed lead to higher fertilization rates [57]. For nearshore echinoderms like sea urchins and sand dollars, water temperature and pH can impact both sperm swimming velocity and fertilization rates (Table 1). Specifically, fertilization rates can show positive, negative, or optimal peak patterns in response to variable temperatures whereas, sperm swimming is generally slower under higher temperatures with some species showing an optimal peak. For both traits, however, studies largely focus on a limited number of thermal treatments preventing accurate estimates of thermal performance curves. Moreover, the combined effects of temperature and pH on sperm swimming and fertilization success remain somewhat equivocal [52]. For tropical urchins in the southern hemisphere, there is contrasting evidence that temperature is more important than pH in affecting fertilization [58], as opposed to work that suggests both pH and temperature are significant determinants of fertilization [59] (Table 1). The degree to which multiple environmental stressors such as temperature and pH influence these reproductive processes appears to be species-specific.

**Table 1:** Effects of temperature and pH on reproductive traits in echinoderms.

Taxon	Location	Temperature (°C)	pH	Response sperm velocity (μm s^-1^)	Response fertilization (%)	Source
SEA URCHINS						
*Mesocentrotus franciscanus*	Vancouver Island, CAN	10	7.55–8.04	- /-	- / ↓	[60]
	California, USA	15	7.26–8.00	- /-	- / ↓	*[61]
*Strongylocentrotus purpuratus*	California/Oregon, USA	14–15	7.61–8.03	- /-	- / ↓	[62]
	California, USA	15	7.26–7.95	- /-	- / ↓	*[61]
*S*. *droebachiensis*	Svalbard, NOR	3–5	7.40–8.31	- /-	- / ↓	[63]
	Arctic	0–8	-	- / -	↓ / -	[64]
*S*. *nudus*	Pohang, KOR	20	6.31–8.03	- / no effect	- / ↓	*[65]
*Hemicentrotus pulcherrimus*	Shimoda, JPN	20	7.59–7.99	- / -	- / no effect	*[65]
	Wakayama, JPN	14	6.83–8.01	- / -	- / ↓	[66]
			7.15–8.3	- / -	- / no effect	
*Echinometra mathaei*	Wakayama, JPN	24	6.79–8.11	- / -	- / ↓	[66]
*E*. *lucunter*	Pedra da Sereia, BRA	26–38	7.4–8.0	- / -	↓ / ↓	[59]
	Florida, USA	6–44	-	- / -	**∩ / -**	[67]
*Lytechinus pictus*	Ireland, GBR	15	7.75–8.07	- / ↓	- / ↓	[68]
*Psammechinus miliaris*	Isle of Cumbrae, GBR	14–20	7.67–8.06	↓ / ↑	- / -	[69]
*Paracentrotus lividus*	Ireland, GBR	14	7.71–8.18	- / ↓	- / -	*[70]
*Pseudoboletia Indiana*	Sydney, AUS	8–25	7.6–8.1	- / -	↑ / ↓	[71]
*Paracihinus angulosus*	Cape Town, ZAF	5–25	-	**∩ / -**	**/ -**	[72]
*Heliocidaris crassispina*	Hong Kong, CHN	28–43	-	- / -	- / ↓	*[73]
*Heliocidaris erythrogramma*	Sydney, AUS	20	7.75–8.07	- / ↑	- / no effect	[68]
SAND DOLLARS						
*Arachnoides placenta*	Queensland, AUS	26	7.12–8.14	- / -	- / ↓	[74]
*Dendraster excentricus*	San Juan Islands, USA	12–22	-	↑ / -	- / -	[56]
	Salish Sea, WA, USA	7–19	-	- / -	no effect / -	[75]
	San Juan Islands, USA	10–20.5	- / -	↑ / -	- / -	[76]
SEA STARS						
*Acanthaster* cf. *solaris*	Guam, FSM	20–36	7.4–8.2	**∩ /** ↓	**∩ /** ↓	[77]
*Heliocidaris erythrogramma*	Sydney, AUS	20–26	7.6–8.17	- / ↓	- / no effect	[58]
	Sydney, AUS	20.5	7.6–8.12	- / no effect	- / no effect	[78]
	Sydney, AUS	-	7.7–8.0	23–26	- / ↓	[79]

The type/shape of response is indicated as positive (↑), negative (↓) or thermal performance curve (**∩**). For response columns, first symbol represents temperature responses and the second pH responses.

* values estimated/digitized from published figure.

In the Northeast Pacific, sand dollar (*Dendraster excentricus*) and red urchin (*Mesocentrotus franciscanus*) distributions extend from Alaska to Baja California and overlap in parts of the Salish Sea [80–82]. Ecologically, red urchins are important in structuring kelp communities through grazing and capture of drift kelp, whereas sand dollars act as ecosystem engineers in infaunal communities through biogenic advection of porewater [83, 84]. Whereas, the effects of temperature on fertilization and sperm swimming have been investigated in these species [75, 76], the limited number of thermal treatments generally reported are not suitable for estimating detailed thermal performance curves. Similarily, the combined effects of acidification and thermal stress has been demonstrated on metabolic rate, gene expression, and larval swimming behavior in these echinoderms, but less is known about the effects on reproductive traits such as fertilization and sperm swimming [22, 85–87].

The waters of the Salish Sea are characterized by outflow of the Fraser River and upwelling from the California Undercurrent that can lead to cool temperatures and persistent aragonite undersaturation [88, 89]. Moreover, pH levels in local surface waters are naturally low (7.86±0.05 [90]; 7.82±0.07 [91]; 7.92±0.30 [92]) and appear to be decreasing rapidly [90, 91, 93, 94]. In the Salish Sea, surface temperatures are predicted to rise 1.5°C over the course of this century [95]. Understanding the consequences of this type of environmental variation on early life history stages is important in predicting the potential impacts of future climate change on marine broadcast spawners. Here, we investigated the effects of variable temperature and pH conditions on sperm swimming and fertilization in two species of echinoderm—sand dollars, *Dendraster excentricus* (Eschscholtz) and red sea urchins, *Mesocentrotus franciscanus* (A. Agassiz, 1863). These data will provide insight into the potential impact that ocean warming and acidification may have on the reproductive ecology of these temperate echinoderms.

## Materials and methods

### Study organisms

Specimens were collected at field sites near the University of Washington-Friday Harbor Laboratories (FHL) on San Juan Island, WA during the summers of 2020 and 2021 (Collection permits held by FHL, not protected species). Sand dollars were collected from shallow beds (<20 cm depth at low tide) at Argyle Lagoon (48–31’12’’ N, 123–00’53’’ W) and sea urchins were collected from tidepools or by snorkel from shallow subtidal habtitats (~2 m depth) at Deadman Bay (48.5353° N, 122.5927° W).

Organisms were maintained at Friday Harbor Laboratories in seatrays supplied with unfiltered, once-through flowing seawater (12.65 ± 0.01°C). Experimental temperatures approximated current and potential future water temperatures at our field sites which were monitored with Bluetooth temperature loggers (HOBO MX2201; Onset, Bourne, MA; temperature logged every 5 or 15 min) to determine the range of conditions experienced.

### Temperature effects on sand dollars and sea urchins

The effects of water temperature on fertilization and sperm swimming performance in both sand dollars and sea urchins was tested in single factor laboratory experiments. A total of 12 temperature treatments were tested for sand dollars (8, 10, 12, 14, 16, 18, 20, 22, 24, 26, 34, and 38°C) and 7 for sea urchins (8, 10, 12, 14, 16, 22°C, and 28°C). These dramatic temperature ranges were designed to capture: 1) the measured field temperatures at each collection site (11.16–33.37°C for sand dollars and 9.68–15.03°C for sea urchins); 2) potential future warming and; 3) the physiological limits of fertilization. For each temperature treatment, gametes were obtained from three independent male-female pairs, each representing a biological replicate (N = 36 unique male-female pairs for sand dollars and 21 unique pairs for sea urchins). Spawning was induced via intracoelomic injections of 1 ml of 0.5M KCl. Eggs were released by inverted females into FSW (salinity = 32.10 ppt, pH = 7.82) for 20–30 minutes, after which eggs were washed 2–3 times. Sperm were kept dry until used and diluted to concentrations of 10^5^ sperm ml^-1^ (estimated via hemocytometer), which was determined to be the optimal concentration based on our preliminary study and previous works (S1 Fig; [76]). To ask whether there are detrimental effects of temperature and pH, we used sperm concentrations that ensure sperm concentrations that neither oversaturate, nor limit gamete concentration permitting a focus on the effects of environmental stressors. Egg solution (1 ml) was mixed with 10 mL of temperature-adjusted water in a 60 × 15 mm Petri-dish ensuring an egg layer no more than two cells thick [64]. 50 μl of diluted sperm solution was added and after 15 minutes [58] fertilization was stopped with the addition of 1 mL of 0.5M KCl to halt sperm motility without inducing additional fertilization [60, 96]. Embryos were incubated in recirculating water baths at appropriate treatment temperatures (Isotemp 4100, Fisher Scientific, Waltham, MA, USA). After 3 hour incubations, development was halted with the addition of 1 mL 4% formaldehyde [60] and fertilization success was determined as the proportion of embryos that had a fertilization membrane or exhibited cleavage [58, 97]. For each male:female pair, fertilization was scored in three 50 embryo subsamples (e.g., three technical replicates = 150 total embryos for each biological replicate).

Sperm swimming performance was also measured under each different temperature. Samples were taken from sperm used in the fertilization experiment and immediately tested for swimming velocity. Specifically, for each male-female pair (e.g., biological replicate), three 3 μl sperm subsamples (e.g., technical replicates) were loaded into a multi-chambered counting slide (20 μm depth; Leja Products, Nieuw-Vennep, Netherlands). Swimming motion was recorded at 60 Hz via a digital camera (20 MP USB 3.0; Amscope, Irvine, CA, USA) mounted on a compound microscope (Olympus BX-40, Center Valley, PA) and recorded via a PC laptop (Alienware 17 R4 or Alienware M17 R3, Dell Technologies, Round Rock, Texas). All sperm in each video recording were tracked for 10 seconds and mean curvilinear velocities were extracted from videos using the *DLTdv digitizing tool*, a MATLAB-based package [98]. Motility was scored as sperm with curvilinear velocities >0 mm s^-1^. For each biological replicate, sperm velocities from three technical replicates were averaged to generate an estimate of sperm swimming velocity. Only sperm that were confirmed to swim continuously in circular patterns were analyzed [99, 100].

### Temperature and pH effects in sand dollars

Fertilization and sperm swimming responses were also measured in sand dollars under different temperatures and pHs. Three pH treatments were set based on calculated target pCO_2_ levels (e.g., 425, 700, 1825 μatm CO_2_ that broadly correlated to pH_NIST_ = 7.9, 7.5 and, 7.1 respectively [101]). These conditions were maintained via CO_2_ bubbled into 1 micron filtered seawater and pH levels were confirmed from water samples taken at the beginning and end of each fertilization trial using a multiparameter water quality meter (HI98194, Hanna Instruments, Woonsocket, RI, USA) calibrated with pH_NIST_ buffers 4, 7, and 10 (Thermo Fisher Scientific). The probe was also checked against an Accumet AB150 pH probe on a bi-weekly basis. Total alkalinity of the lab seawater supply was measured via titration six times throughout the experiment and also spot checked with a seawater alkalinity colorimeter every 1–2 days (Hanna HI772).

Three experimental water temperatures of 8, 16, and 24°C were maintained by incubating 35 × 10 mm petri dishes (Corning, Glendale, AZ) in recirculating chillers. This resulted in a total of nine temperature × pH treatments. Gametes from each male:female pair (e.g., each biological replicate) were tested under one temperature but across all three pH treatments (see S1 Table). For each biological replicate, fertilization success was averaged from three technical replicates of 50 eggs each. For sperm swimming performance, three 3 ul sperm samples were averaged as technical replicates for each trial as described above.

### Statistical analyses

We evaluated the effects of temperature on fertilization rate, sperm swimming velocity, and the percent sperm motile using a non-linear approach described by Padfield et al. [102]. For each variable, we fitted 10 TPC models (S2 and S3 Tables) using non-linear least squares with the R package *rTPC* and bootstrapping for 1000 iterations. For each dataset, optimal fit was determined as the model with the lowest Akaike Information Criterion (AICc) corrected for small sample sizes [103]. From the optimal model, several derived TPC parameters were estimated: maximum rate (r_max_); optimum temperature (T_opt_) as the temperature where maximum rate is achieved and; thermal breadth (T_br_) as the range of temperatures at which rates exceed 80% of the rate at T_opt_. Uncertainty in TPC parameter estimates was assessed with bootstrap (N = 5000) confidence intervals for each parameter.

In the second experiment examining multiple stressors, variation in the three reproductive measures (e.g., fertilization, swimming velocity, motility) was assessed. Where the assumptions of the general linear model failed, we employed a nonparametric aligned rank transform (ART) ANOVA procedure [104]. This nonparametric method addresses detection of interaction effects in factorial designs. Analyses were performed with the R stats package “ARTool” [105] (RStudio, 2021.09.1 Build 372) and pairwise differences were assessed with Bonferroni adjusted posthoc tests. Effect sizes were estimated for each independent variable (partial eta squared; η^2^) and also for each pairwise comparison (Cohen’s d) and then classified according to [106–108].

### Fertilization kinetics model

To assess the potential effects of sperm swimming on fertilization rates, we estimated fertilization for the conditions used in our experiments using a fertilization kinetics model [97, 109]. Specifically, we used our model to explore the notion that extreme temperature and pH conditions alone can slow swimming speeds enough to lead to the lower fertilization rates that we observed in our experiments. The proportion of fertilized eggs (φ) was calculated as,

$$\varphi =1-exp\left(-\frac{\beta S_{0}}{\beta_{0}E_{0}}(1-e^{-\beta_{0}E_{0}t})\right)$$ (1)

where S_0_ is sperm concentration (number of sperm μl^-1^), E_0_ is egg concentration (number of eggs μl^-1^), t is time of egg exposure to sperm (s), β is the fertilization rate constant of fertilization (mm^3^ s) and β_0_ is the rate constant for egg-sperm contact. β/β_0_ ratios were estimated by fitting our data with nonlinear regression and then compared for agreement with empirical estimates of β_0_ that were calculated as,

$$\beta_{0}=\upsilon \times \sigma_{0}$$ (2)

where υ is sperm velocity (μm s^-1^) and σ_0_ is egg cross-sectional area (μm^2^).

## Results

Water temperatures were generally higher and showed a wider range at the field site where sand dollars were collected compared to where sea urchins were collected in 2020 and 2021 (Argyle Lagoon = 19.22±0.07°C versus Deadman Bay = 11.76±0.03°C).

### Effects of temperature on sand dollars

**Fig 1:**
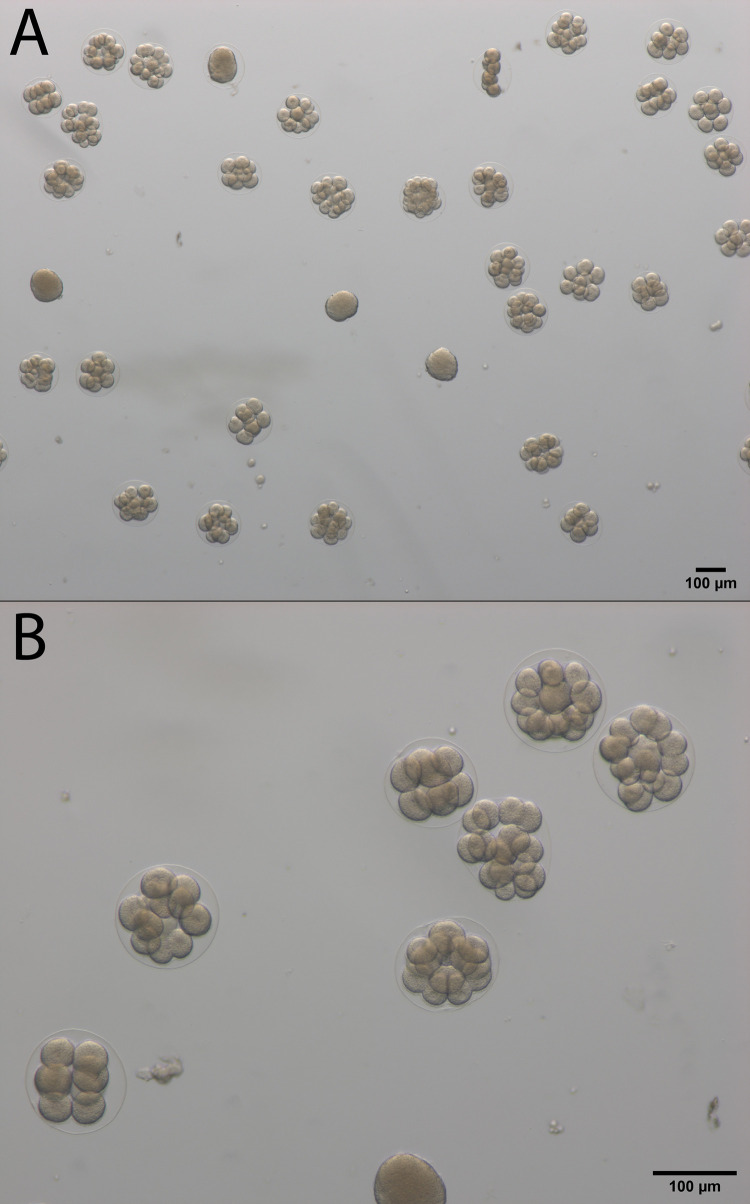
Sand dollar embryos 3 hours post-fertilization. (A). fertilized and unfertilized embryos at 50× magnification. (B). Close up at 112× magnification highlighting fertilization membrane. Z-stack images were captured using an Axio Zoom V16 stereo microscope equipped with an Axiocam 506 color camera (Zeiss, Germany).

Successful fertilization occurred in all treatments indicated by cell division and/or the presence of a fertilization envelope (Fig 1A and 1B).

**Fig 2:**
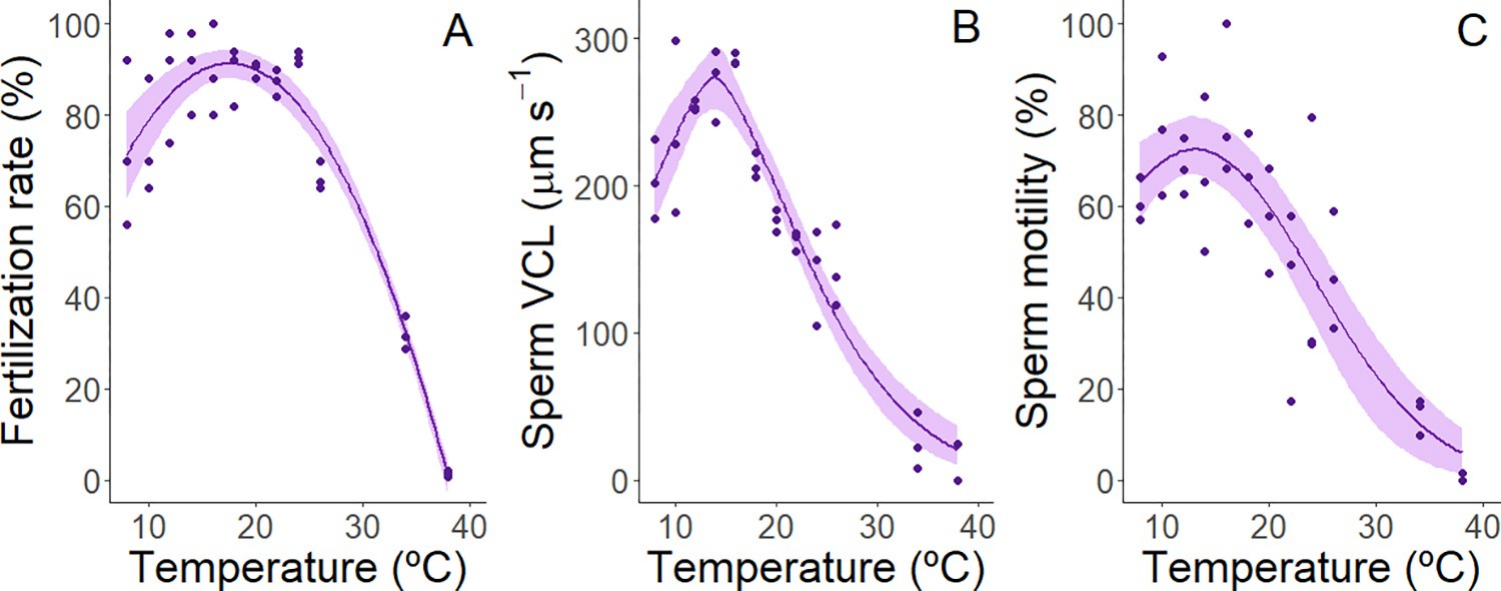
Effect of water temperature on reproductive traits of sand dollars, *Dendraster excentricus*. (A). fertilization rates, (B). sperm curvilinear velocity, and (C). proportion of sperm that were motile. Data for the three traits were fitted with non-linear functions (quadratic, modified gaussian and gaussian respectively) and plotted with 95% confidence intervals (shaded areas) determined by residual bootstrapping [102].

In sand dollars, fertilization success remained high (e.g., mean fertilization > 87%) over a wide range of temperatures from 12 to 24°C (Fig 2A). Rates were lower at cooler temperatures down to 8°C (73%) and warmer temperatures up to 38°C (~1%). Sperm swimming velocities increased from 8 to 16°C and then decreased markedly as temperatures approached 38°C, where velocities were nearly zero (Fig 2B). Similarly, the proportion of sperm that were motile rose from 61 to 81% over temperatures from 8–18°C and then dropped dramatically at higher temperatures (Fig 2C).

### Effects of temperature on sea urchins

**Fig 3:**
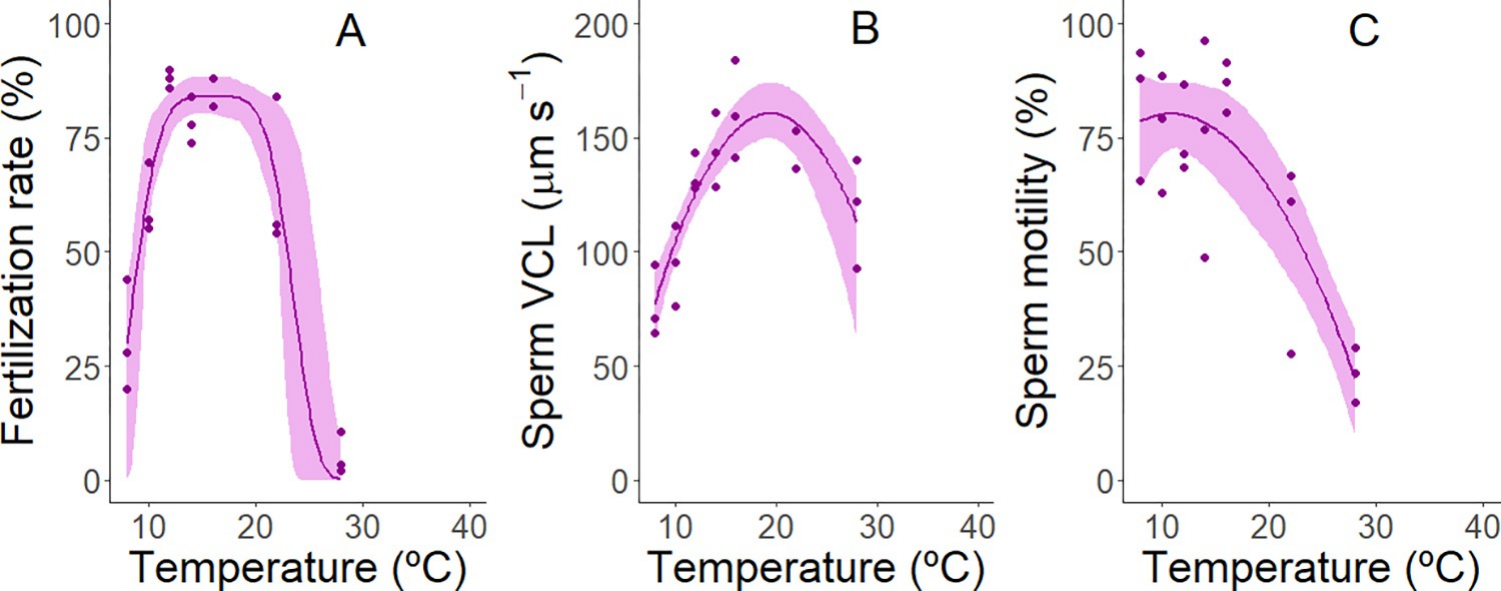
Effect of water temperature on reproductive traits of red urchins, *Mesocentrotus franciscanus*. (A). fertilization rates (B). sperm swimming velocity, and (C). proportion of sperm that are motile. Data were fitted with non-linear functions (modified gaussian for fertilization, quadratic for sperm velocity and motility) and plotted with 95% confidence intervals (shaded areas) determined by residual bootstrapping [102].

For red urchins, fertilization was highest at temperatures between 12 and 22°C (mean fertilization >64%; Fig 3A). Rates were markedly reduced at cooler temperatures down to 8°C (31%), and warmer temperatures up to 28°C (5%). Sperm velocity peaked at 16°C (Fig 3B), whereas sperm motility remained relatively high from 8 to 16°C (e.g., >74%) and only decreased at higher temperatures (Fig 3C).

### Estimated parameters

**Fig 4:**
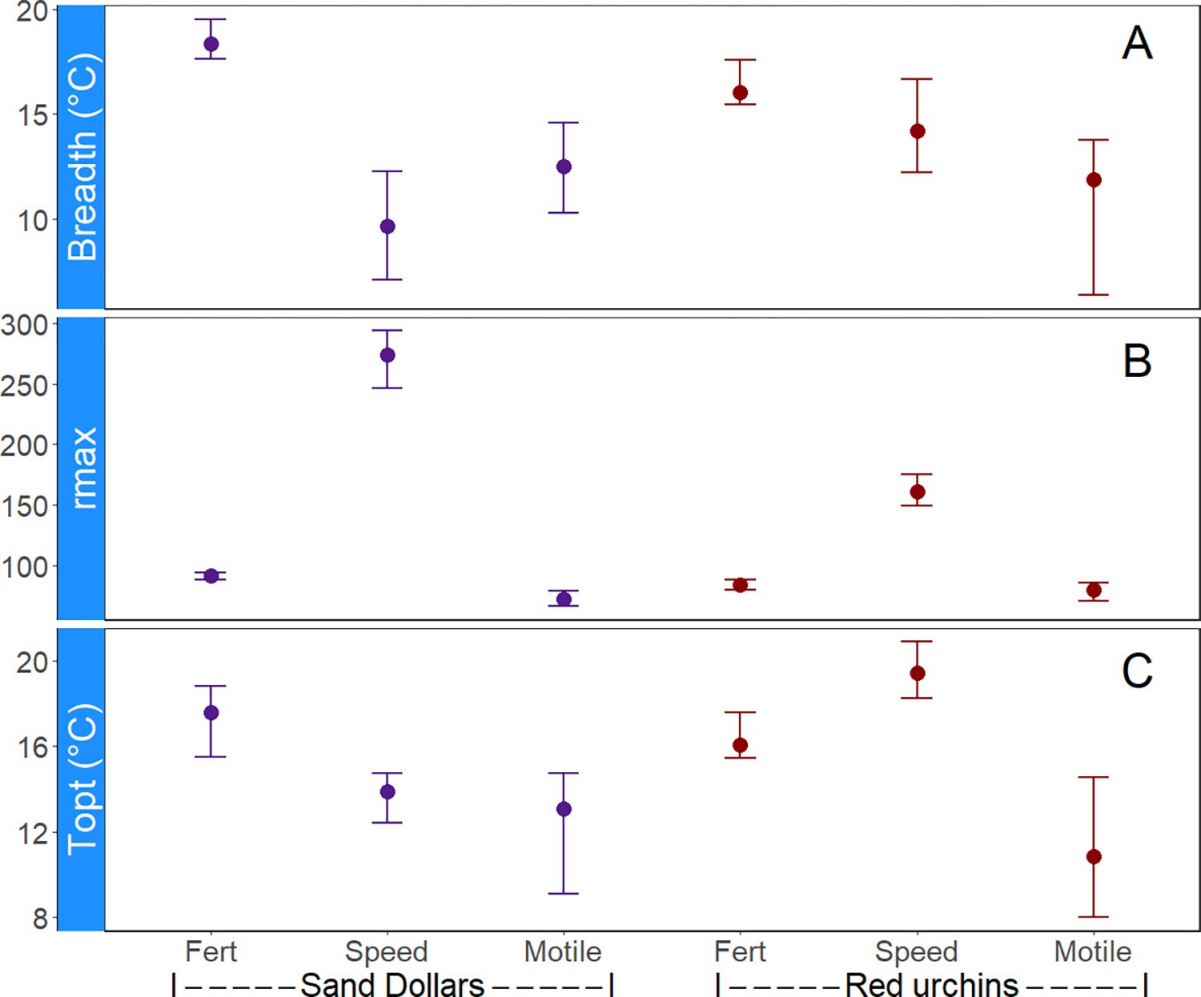
Estimates for derived parameters from thermal performance curves of sand dollar and sea urchin reproductive traits. Reproductive parameters include r_max_ = maximum rate, T_opt_ = thermal optimum, and Breadth = thermal breadth in panels A-C respectively. Error bars represent the 95% bootstrap confidence intervals.

Maximum sperm swimming velocity was significantly faster in sand dollars compared to red sea urchins (Fig 4A; S4 Table). For sand dollars, thermal breadth (T_br_) and optimal temperatures (T_opt_) were higher for fertilization rates than for sperm swimming velocity and motility (Fig 4B and 4C). Similarly, in red urchins thermal breadth was higher for fertilization compared to sperm swimming or motility. However, fertilization rates in urchins had a lower optimal temperature than sperm swimming, but higher than motility.

### Combined effects of temperature and pH on sand dollars

**Fig 5:**
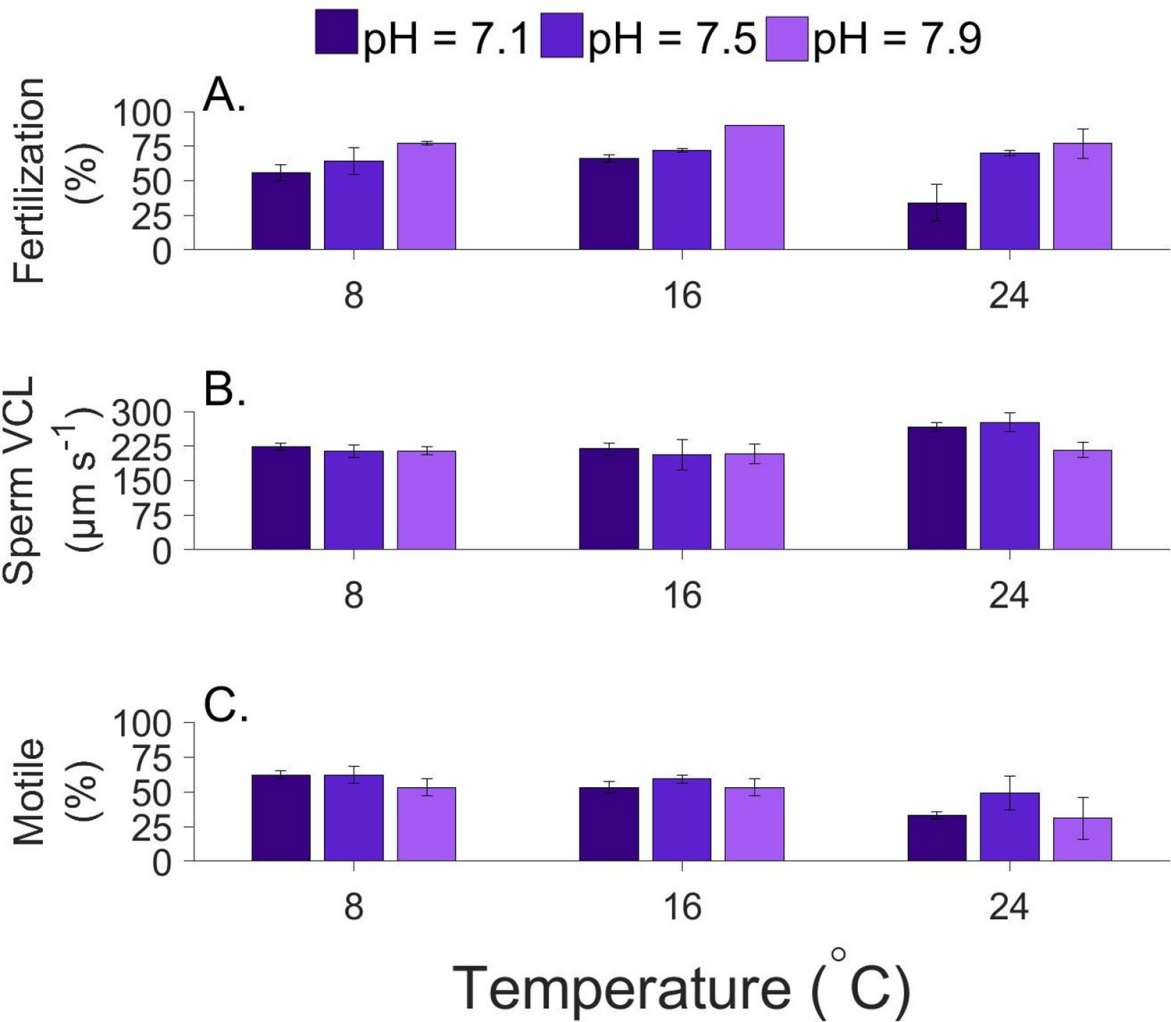
Combined effects of temperature and pH on reproductive traits of sand dollars, *Dendraster excentricus*. (A). fertilization rate, (B). sperm curvilinear velocity and, (C). sperm motility. N = 3 adult pairs. Error bars represent 1 SE.

Fertilization rates for sand dollars ranged from 10 to 91% and was highest at moderate temperature (16°C) and highest pH (7.9) conditions. Fertilization was significantly affected by both water temperatures (Fig 5A; F_(2,18)_ = 5.607, p = 0.042), and pH (F_(2,18)_ = 17.1935, p < 0.001) with both being large effects (partial η^2^ = 0.451 and 0.628 respectively). Although there was no statistically significant interaction between pH and temperature (F_(4,18)_ = 1.146, p = 0.367), the largest change occurred at the lowest pH (between 7.1 and 7.5) and the highest temperature (24°C). Pairwise post hoc tests indicate that fertilization was significantly higher at 16°C compared to 24°C and that this was a large effect (Cohen’s d = 1.812). For pH, differences in fertilization were between pH = 7.1 and 7.5 (large effect; Cohen’s d = 1.152), pH 7.5 and 7.9 (very large effect; Cohen’s d = 1.440) and pH = 7.1 and 7.9 (huge effect; Cohen’s d = 2.592).

Swimming velocities ranged from 159 to 370 μm s^-1^ and were significantly affected by water temperatures (Fig 5B; F_(2,18)_ = 5.391, p = 0.016; partial η^2^ = 0.319 is a large effect), but not pH (F_(2,18)_ = 2.019, p = 0.165). Post hoc tests indicate that velocities were significantly faster at 24°C compared to 8 and 16°C and were “large” and “very large” effects respectively (Cohen’s d = 1.07 and 1.27). More specifically, the largest difference in velocity occurred at 24°C between pH = 7.9 where velocities were slow (231 μm s^-1^) and pH = 7.1 and 7.5 where velocities were elevated (284 and 295 μm s^-1^ respectively).

The proportion of sperm that were motile ranged from 33 to 62% and was significantly affected by water temperature (Fig 5C; F_(2,18)_ = 6.680, p = 0.008; partial η^2^ = 0.433 is a large effect), but not pH (F_(2,18)_ = 2.131, p = 0.151). Post hoc tests indicate that sperm motility at 24°C was significantly lower than 8°C, representing a large effect (Cohen’s d = 1.647). Although the pH effect was not statistically significant, differences among pH conditions were most pronounced at 24°C.

### Measured versus model estimates of fertilization

**Fig 6:**
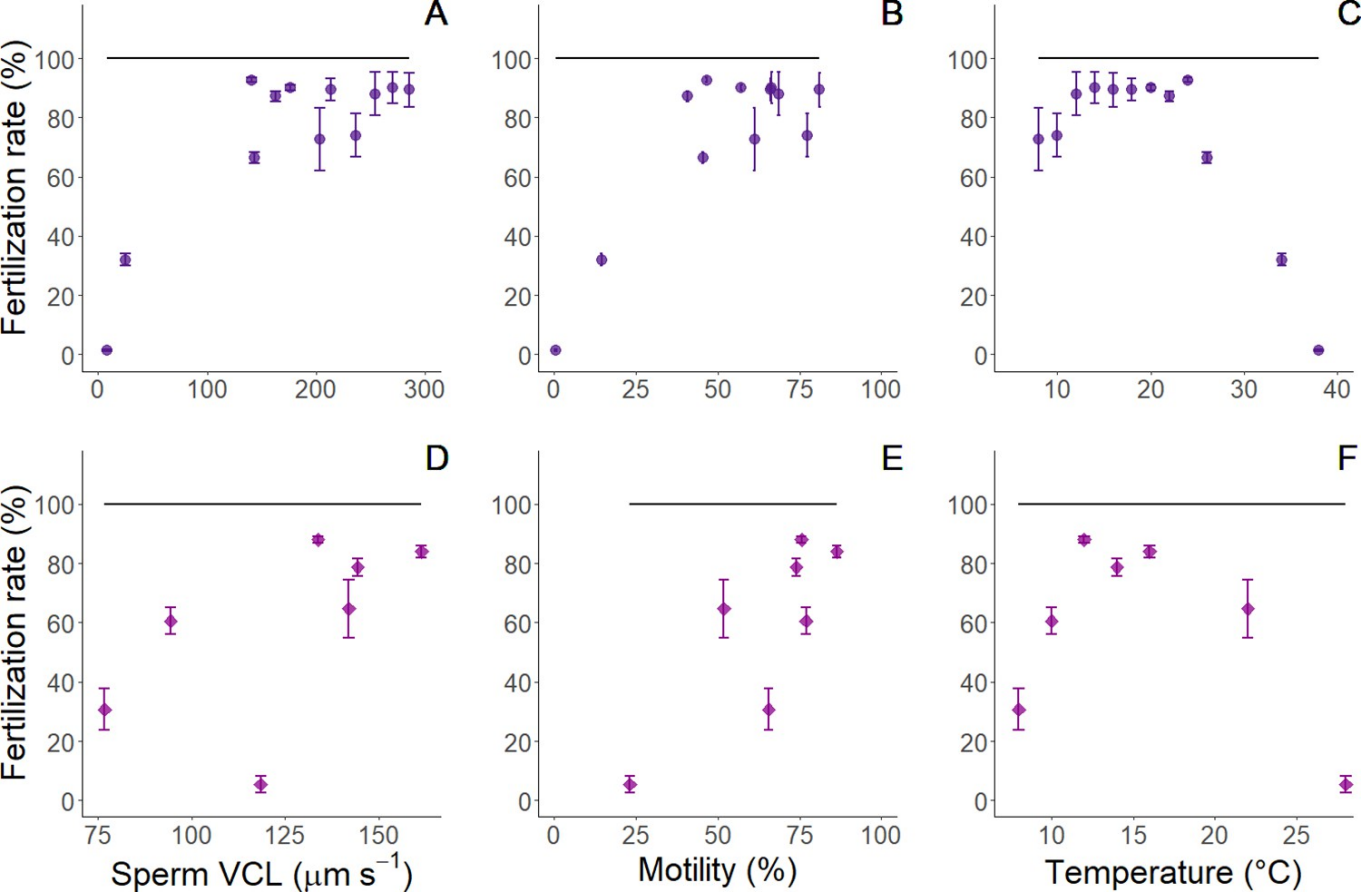
Comparison of empirical and modelled fertilization rates in single factor experiments. Fertilization responses to sperm swimming velocity (panels A, D), motility (panels B, E) and water temperatures (panels C, F). Top row are for sand dollars and bottom row are for sea urchins. Lines represent estimates from fertilization kinetics model [97, 109] parameterized with swimming velocities and symbols represent empirical fertilization rates from this study.

For single factor experiments, measured sand dollar fertilization rates were positively correlated with both swimming velocity and motility (Spearman’s ρ = 0.474, p = 0.003; Spearman’s ρ = 0.480, p = 0.003 respectively; symbols in Fig 6). In sea urchins, fertilization rates positively correlated with velocity, but not motility (Spearman’s ρ = 0.537, p = 0.012 and Spearman’s ρ = 0.334, p = 0.139 respectively). Model estimates predicted full fertilization for all conditions and all swimming velocities in our experiments (Fig 6C and 6D). Modelled fertilization rates (black lines in Fig 6) were higher than measured fertilization for all treatments.

**Fig 7:**
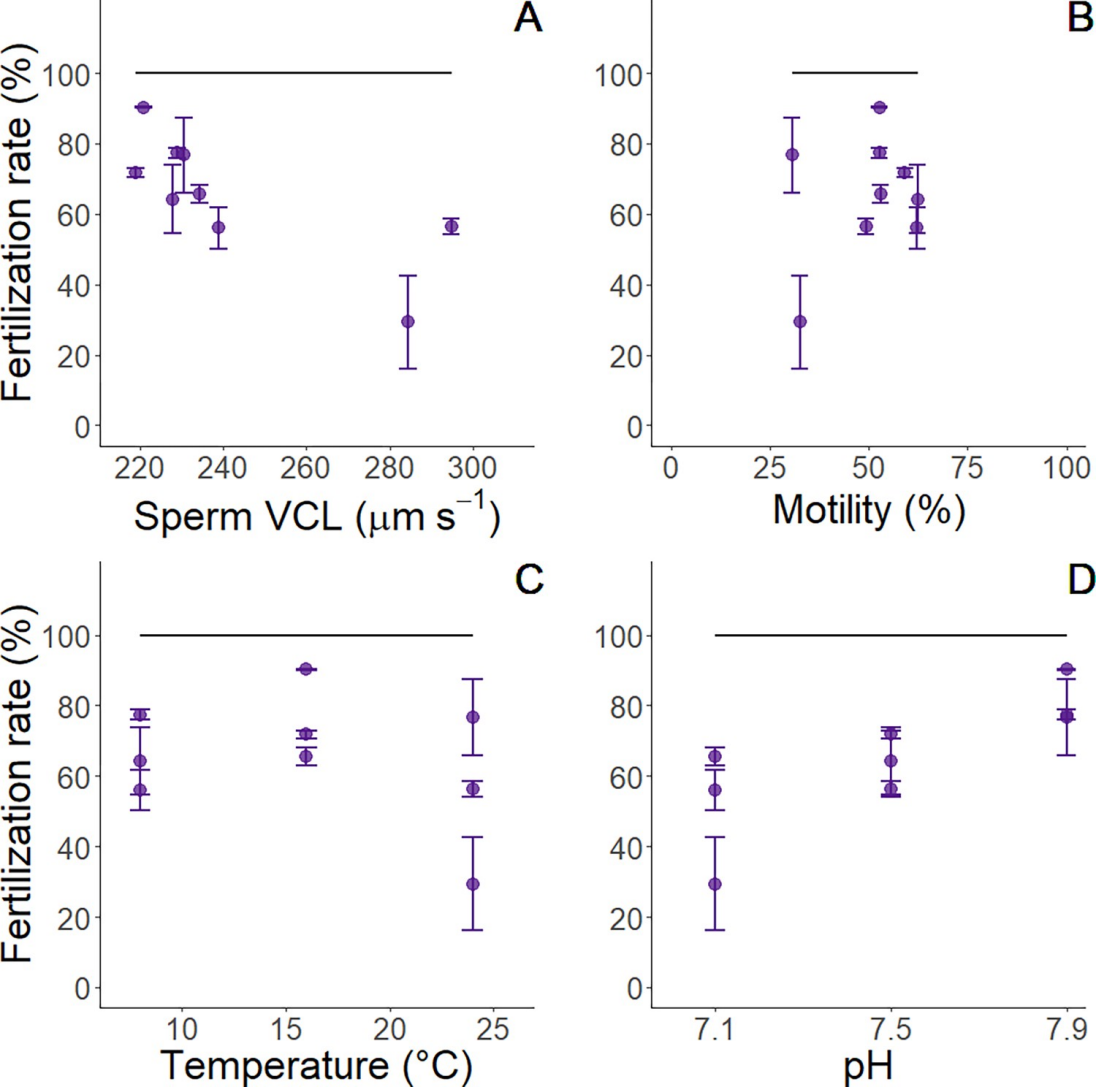
Comparison of empirically measured versus modelled sand dollar fertilization rates. Fertilization responses to: (A). sperm swimming velocity; (B). sperm motility; (C). water temperature and; (D) pH. Lines represent estimates from fertilization kinetics model parameterized with swimming velocities and symbols represent empirical fertilization rates from this study [97, 109].

In our temperature × pH experiments, fertilization rates decreased with increasing sperm velocity (Fig 7A) and the correlation was significant (Spearman’s ρ = -0.420, p = 0.029). In contrast, motility and fertilization rates were not significantly correlated (Spearman’s ρ = -0.143, p = 0.477). Modelled fertilization rates were 100% across all trials and significantly higher than all corresponding measured fertilization rates.

## Discussion

Variation in seawater temperature is characteristic of many nearshore ecosystems. Our results, covering a wide range of temperatures, showed responses that were consistent with TPCs reported for many traits from a wide array of species [110–114]. Although simple positive or negative responses to increasing temperatures have been reported in some echinoderms (Table 1), these results typically emerge from experiments covering a narrower thermal range than tested here. In studies that use a similarly wide set of temperature treatments (e.g., ≥20°C range), nonlinear TPC-shaped responses are observed. For instance, although fertilization in warm water urchins from Florida, USA and sea stars from Australia display higher optimal temperatures (27–34°C vs. 24-32°C respectively), they also display nonlinear TPC shaped response curve when tested across wide temperature ranges [67, 77]. Indeed, this is consistent with the notion that when tested over a wide range of temperatures (20–35°C), nonlinear TPC-shaped functions are more appropriate compared to simple linear fits.

In sand dollars, the thermal optimum for fertilization rates was higher than previously reported. Our specimens were collected during the late spring-early summer months, when measured water temperatures at the sand dollar site exceeded 30°C on hot days, which may help explain why our T_opt_ was higher than previously reported (14°C) for the same region [76]. The similarity of optimal temperatures for fertilization rates in both sand dollars and sea urchins is notable given the stark differences in thermal conditions at the collection sites for each species. Whereas, T_opt_ for sand dollars (17.6°C) was close to mean temperatures experienced during the spring/early summer (19.22±0.07°C), sea urchin T_opt_ (16.7°C) was higher than the mean water temperatures in the field (e.g., 11.76±0.03°C). Although T_opt_ may be influenced by regional rather than local temperatures, at our sites sand dollars appear to be living closer to their thermal limits than red sea urchins.

Our results also suggest that sand dollars are tolerant to a wider range of temperatures compared to red sea urchins as evidenced by broader thermal breadths in fertilization rate (18.33°C vs. 16.04°C) and sperm swimming VCL (14.18°C vs. 9.66°C; see Fig 4B and S4 Table). Although our estimates of sand dollar sperm velocity were similar to previous reports, they peaked at a lower temperature (13.9°C) compared to previous work showing velocities increasing up to 26°C [76]. Moreover, sand dollar T_opt_ for swimming velocity and motility (~13°C) were much lower than mean temperatures measured at the field site during collection (2020 = 19.0°C, 2021 = 17.8°C), daily tidal fluctuations dropped water temperatures to 12-13°C, much closer to T_opt_ for both traits. For red urchins, our estimates of velocity are lower than previously reported (77 and 94 μm s^-1^ at 8 and 10°C in our experiments versus 130 μm s^-1^ at 9°C from previous work [97]). It should be noted, however, that velocities in our experiment rose rapidly at 12°C (e.g., 134 μm s^-1^), suggesting that the discrepancy could be related to sensitive physiological thresholds in the two populations.

Our measures of swimming were made on sperm that had not been exposed to eggs. Indeed, sperm swimming activity and/or velocity increases in the presence of chemoattractants for a number of sea urchin species [115, 116]. Although sperm generally swim in stereotypical circular patterns, they also move towards chemical cues by tracing concentration gradients [117, 118]. Such changes in behavior potentially affect swimming and fertilization responses to environmental stress and await future investigation. Beyond single-factor effects, our results also quantify responses to multiple environmental stressors. In sand dollars, fertilization rates were lower at higher temperatures and lower pH (Fig 5). In Australian sea urchins, *Heliocidaris erythrogramma* and *Pseudoboletia indiana*, temperature, but not pH affected sea urchin fertilization [58, 71, 78]. However, work from single-factor experiments showed that lower pH (7.7 vs 8.1 pH) can also impede fertilization success in *Heliocidaris erythrogramma* and purple sea urchins (*Strongylocentrotus purpuratus*) [62, 79]. Although we detected effects of both temperature and pH on sand dollar fertilization, we did not observe an interactive effect as has been reported in Antarctic sea urchins [119]. Moreover, our results add to the notion that both temperature and pH can reduce fertilization success.

For sand dollars, both sperm swimming velocity and percent motility were affected by temperature whereas, pH did not. This is consistent with previous single-factor work in echinoids showing that temperature influences sperm velocity and motility [72], but variable pH does not [65]. Although low pH conditions lead to faster sperm swimming velocities in some sea urchin species [68, 69], more commonly, low pH leads to slower and/or less motile sperm [70, 79, 115]. In one Australian sea urchin, pH (7.6–8.1) was important in reducing sperm motility, but not velocity [78]. These differences may be, in part, due to the low and variable pH conditions found in the Salish Sea, a notion that warrants further investigation.

Our model estimates predicted 100% fertilization rates over the entire range of sperm swimming velocities in our experiments. This result is consistent with previous work on red urchins that showed similarly high modelled fertilization rates using similar sperm and egg concentrations as used in our experiments [120]. Previous work, however, used ideal (max or near max) sperm swimming velocities when modeling fertilization, whereas our experiments demonstrate that sperm swimming velocities slow, sometime markedly under extreme temperature or pH conditions. Our model predicts that the slow sperm velocities observed in our experiments should have little/no effect on fertilization rate. This result stands in contrast to our observed fertilization rates that were lower at extreme temperatures or pH. Moreover, the discrepancy between modelled and measured fertilization rates was most pronounced under extreme temperature and pH conditions. These results suggest potential impairment of other mechanisms involved with aspects of the reproductive process beyond simple sperm swimming (e.g., chemoattraction, sperm receptors on the egg) [116, 121].

In this study, we investigated how environmental conditions impact the reproductive performance of sea urchins and sand dollars. Results showed wider thermal breadths in sand dollars relative to urchins, perhaps reflecting the wider range of water temperatures experienced by sand dollars at our field sites. Whereas, sand dollar fertilization was affected by both temperature and pH, only temperature influenced sperm swimming. A fertilization kinetics model parameterized with our swimming data dramatically overestimated measured fertilization rates and this discrepancy was most pronounced under extreme temperature and pH conditions. Moreover, our results suggest that environmental stressors like temperature and pH likely impair aspects of the reproductive process beyond simple sperm swimming behavior.

## Supporting information

S1 Fig Water temperatures from collection sites of sand dollars and sea urchins used in fertilization experiments. Panel A represents temperatures from our sand dollar collection site (Argyle Creek, WA, USA; N = 4908) and panel B represents our sea urchin collection site (Deadman’s Bay, WA, USA; N = 4497).
(TIF)

S2 Fig Standard curve of fertilization rates as a function of sperm concentrations in red urchins, *Mesostrongylocentrotus franciscanus*. Gametes are from one male: female pair and symbols represent means±SE of three subsamples at each concentration. Concentrations estimated from hemocytometer counts.
(TIF)

S1 Table Experimental design for temperature × pH fertilization experiment with sand dollars. Hatched arrows indicate males used across all pH treatments.
(DOCX)

S2 Table List of models fitted for thermal performance curves. (DOCX)

S3 Table Fitted thermal performance curve models. Models ranked by small-sample corrected Akaike Information Criterion (AICc) and Bayesian Information Criterion (BIC) values for each reproductive trait.
(DOCX)

S4 Table Parameter estimates with 95% confidence intervals from thermal performance curves of sand dollars and sea urchins. T_opt_ = optimum temperature (°C); T_br_ = thermal breadth (°C) and; r_max_ = maximum rate. Parameters that differ significantly between acclimation treatments are shown in bold.
(DOCX)
