# Supplementary figures and images for: The effects of temperature and pH on the reproductive ecology of sand dollars and sea urchins: Impacts on sperm swimming and fertilization
Source: PLoS One. 2022 Dec 1;17(12):e0276134. doi: 10.1371/journal.pone.0276134 (PMC9714736; doi:10.1371/journal.pone.0276134)

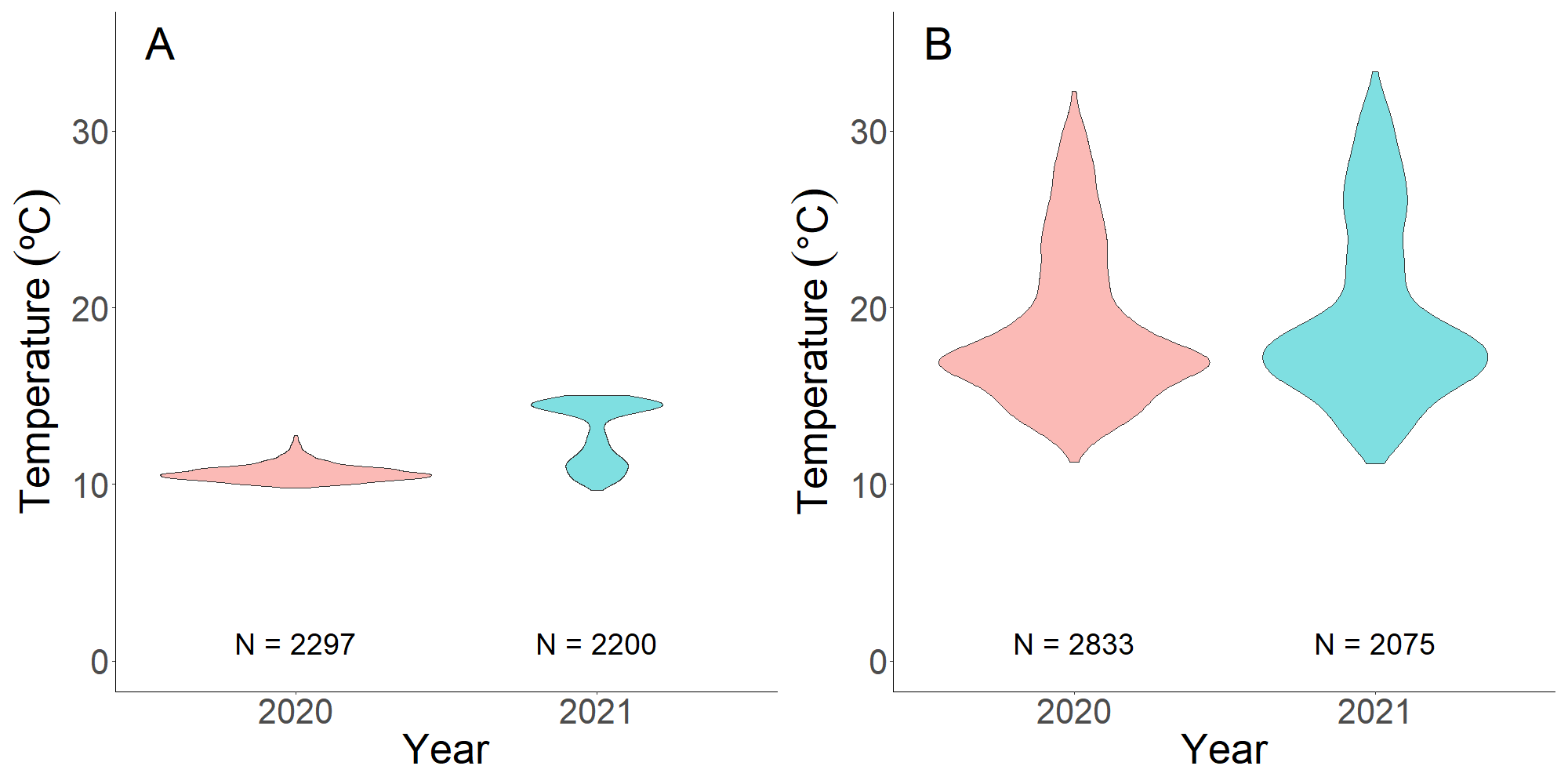

Supplement: S1 Fig — Panel A represents temperatures from our sand dollar collection site (Argyle Creek, WA, USA; N = 4908) and panel B represents our sea urchin collection site (Deadman’s Bay, WA, USA; N = 4497). (TIF) [file pone.0276134.s001.tif]

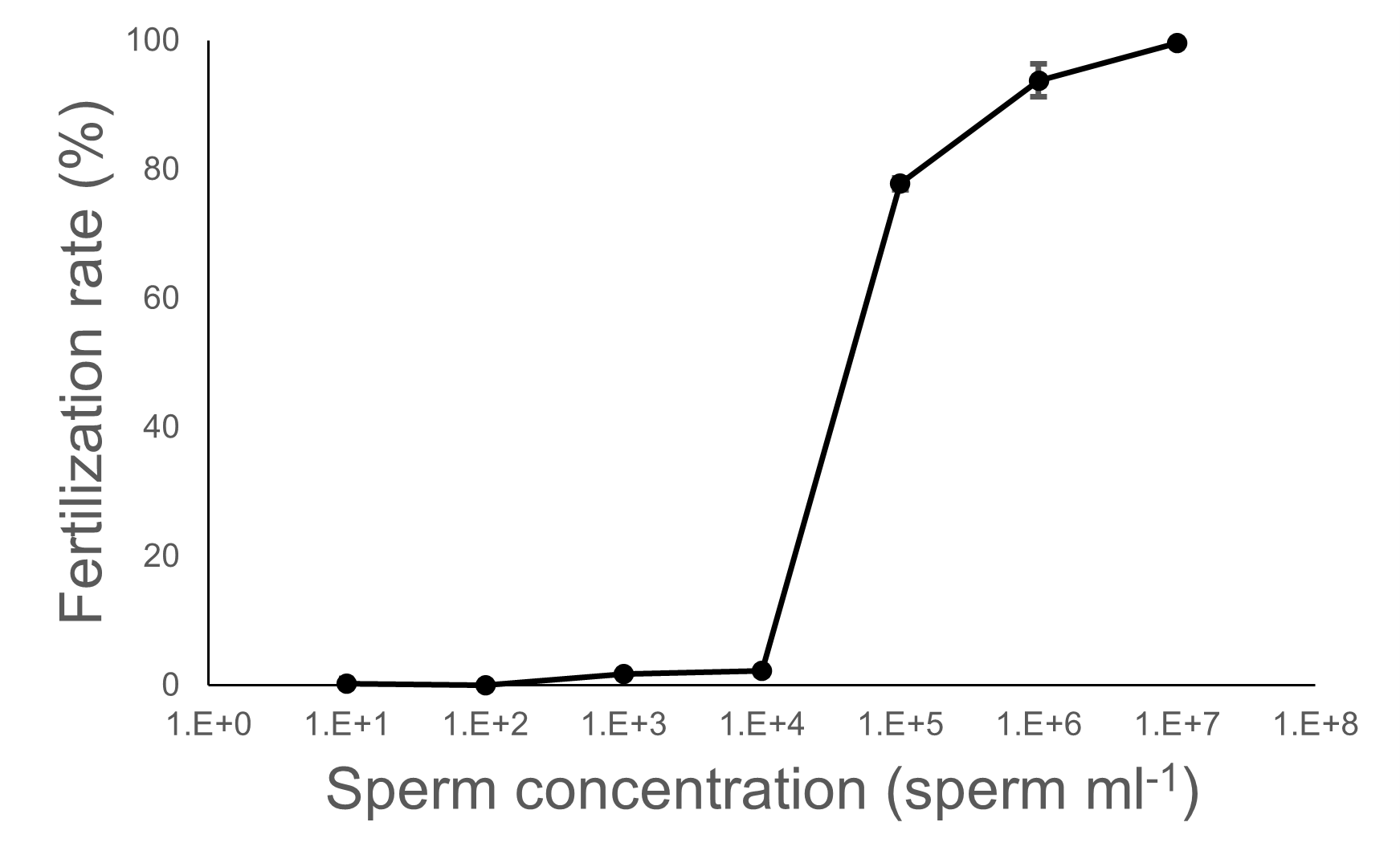

Supplement: S2 Fig — Gametes are from one male: female pair and symbols represent means±SE of three subsamples at each concentration. Concentrations estimated from hemocytometer counts. (TIF) [file pone.0276134.s002.tif]
