# Supplementary material for: The effects of temperature and pH on the reproductive ecology of sand dollars and sea urchins: Impacts on sperm swimming and fertilization
Source: PLoS One. 2022 Dec 1;17(12):e0276134. doi: 10.1371/journal.pone.0276134 (PMC9714736; doi:10.1371/journal.pone.0276134)
Supplement: S1 Table — Hatched arrows indicate males used across all pH treatments. (DOCX) [file pone.0276134.s003.docx]

**S1 Table.** Experimental design for temperature × pH fertilization experiment with sand dollars. Hatched arrows indicate males used across all pH treatments.

| Temperature (°C) |  | pH | | |
| --- | --- | --- | --- | --- |
|  |  | 7.1 | 7.5 | 7.9 |
| 8 |  | Female #1 | Female #2 | Female #3 |
|  | Male #1 -----------------------------------------------------------------> | | | |
| 8 |  | Female #4 | Female #5 | Female #6 |
|  | Male #2 -----------------------------------------------------------------> | | | |
| 8 |  | Female #7 | Female #8 | Female #9 |
|  | Male #3 -----------------------------------------------------------------> | | | |
| 16 |  | Female #10 | Female #11 | Female #12 |
|  | Male #4 -----------------------------------------------------------------> | | | |
| 16 |  | Female #13 | Female #14 | Female #15 |
|  | Male #5 -----------------------------------------------------------------> | | | |
| 16 |  | Female #16 | Female #17 | Female #18 |
|  | Male #6 -----------------------------------------------------------------> | | | |
| 24 |  | Female #19 | Female #20 | Female #21 |
|  | Male #7 -----------------------------------------------------------------> | | | |
| 24 |  | Female #22 | Female #23 | Female #24 |
|  | Male #8 -----------------------------------------------------------------> | | | |
| 24 |  | Female #25 | Female #26 | Female #27 |
|  | Male #9 -----------------------------------------------------------------> | | | |
