# Supplementary material for: The effects of temperature and pH on the reproductive ecology of sand dollars and sea urchins: Impacts on sperm swimming and fertilization
Source: PLoS One. 2022 Dec 1;17(12):e0276134. doi: 10.1371/journal.pone.0276134 (PMC9714736; doi:10.1371/journal.pone.0276134)
Supplement: S2 Table — (DOCX) [file pone.0276134.s004.docx]

**Table S2.** List of models fitted for thermal performance curves.

| **Model** | **Equation** | **Parameters** | **Reference** |
| --- | --- | --- | --- |
| Beta | *rate = (a.((T - b + ((c.(d-1))/(d + e - 2)))/c)^(d-1).(1 - ((T - b + ((c.(d-1))/(d + e - 2)))/c))^(e-1)) / (((d-1)/(d + e - 2))^(d-1).((e-1)/(d + e - 2))^(e-1))* | **a, b, c, d, e**: dimensionless parameters | [1] |
| Boatman | *rate = rmax.(sin(pi.((temp - tmin)/(tmax - tmin))^a))^b* | **rmax, tmin, tmax, a, b** | [2] |
| Delong | *rate = c.exp(-(eb-(ef.(1-((temp + 273.15)/tm))+ehc.((temp + 273.15)-tm-((temp + 273.15).log((temp + 273.15)/tm)))))/(k.(temp + 273.15)))* | **c, eb, ef, tm, ehc** | [3] |
| Flinn | *rate = 1 / (1 + a + b.T + c.T^2)* | **a**: controls the height of the curve  **b**: controls the slope of the initial increase of the curve  **c**: controls the position and steepness of the decline of the curve | [4] |
| Gaussian | *rate = rmax.exp(-0.5.(abs(T - topt)/a)^2)* | **rmax**: maximum rate at optimum temperature  **topt**: optimum temperature (ºC)  **a**: related to the full curve width | [5] |
| Jöhnk | *rate = rmax.(1 + a.((b^(temp - topt) - 1) - (log(b)/log(c)).(c^(temp - topt) - 1)))* | rmax, topt, a, b, c | [6] |
| Modified Gaussian | *rate = rmax.exp(-0.5.(abs(T - topt)/a)^b)* | **rmax**: maximum rate at optimum temperature  **topt**: optimum temperature  **a**: related to full curve width  **b**: allows for asymmetry in the curve fit | [7] |
| Quadratic | *rate = a + b.T + c.T^2* | **a**: parameter that defines the rate at 0 ºC  **b,c**: parameters with no biological meaning | [8] |
| Spain | *rate = est = r0. exp(a.T) . (1 - b.exp(c.T))* | **a**: constant that determines the steepness of the rising portion of the curve  **b**: constant that determines the position of topt  **c**: constant that determines the steepness of the decreasing part of the curve  **r0**: the apparent rate at 0 ºC | [9] |
| Thomas | *rate = a . exp(b . temp) - (c + d.(exp(e.temp)))* | **a, b, c, d, e** | [10] |
| Weibull | *rate = ((a.(((c-1)/c)^((1-c)/c)).((((T-topt)/b)+(((c-1)/c)^(1/c)))^(c-1)).(exp(-((((T-topt)/b)+(((c-1)/c)^(1/c)))^c)+((c-1)/c)))))* | **a**: scale the height of the curve  **topt**: optimum temperature  **b**: defines the breadth of the curve  **c**: defines the curve shape | [7] |

1. Niehaus AC, Angilletta MJ Jr, Sears MW, Franklin CE, Wilson RS. Predicting the physiological performance of ectotherms in fluctuating thermal environments. Journal of Experimental Biology. 2012;215: 694–701. doi:10.1242/jeb.058032

2. Boatman TG, Lawson T, Geider RJ. A key marine diazotroph in a changing ocean: the interacting effects of temperature, CO_2_ and light on the growth of *Trichodesmium erythraeum* IMS101. PLOS ONE. 2017;12: e0168796. doi:10.1371/journal.pone.0168796

3. DeLong JP, Gibert JP, Luhring TM, Bachman G, Reed B, Neyer A, et al. The combined effects of reactant kinetics and enzyme stability explain the temperature dependence of metabolic rates. Ecology and Evolution. 2017;7: 3940–3950. doi:10.1002/ece3.2955

4. Flinn PW. Temperature-dependent functional response of the parasitoid *Cephalonomia waterstoni* (Gahan) (Hymenoptera: Bethylidae) attacking rusty grain beetle larvae (Coleoptera: Cucujidae). Environmental Entomology. 1991;20: 872–876. doi:10.1093/ee/20.3.872

5. Lynch M, Gabriel W. Environmental tolerance. The American Naturalist. 1987;129: 283–303. doi:10.1086/284635

6. Jöhnk KD, Huisman J, Sharples J, Sommeijer B, Visser PM, Stroom JM. Summer heatwaves promote blooms of harmful cyanobacteria. Global Change Biology. 2008;14: 495–512. doi:10.1111/j.1365-2486.2007.01510.x

7. Angilletta MJ. Estimating and comparing thermal performance curves. Journal of Thermal Biology. 2006;31: 541–545. doi:10.1016/j.jtherbio.2006.06.002

8. Montagnes DJS, Morgan G, Bissinger JE, Atkinson D, Weisse T. Short-term temperature change may impact freshwater carbon flux: a microbial perspective. Global Change Biology. 2008;14: 2823–2838. doi:10.1111/j.1365-2486.2008.01700.x

9. Spain JD. BASIC microcomputer models in biology. Reading, MA (USA) Addison-Wesley; 1982.

10. Thomas MK, Aranguren-Gassis M, Kremer CT, Gould MR, Anderson K, Klausmeier CA, et al. Temperature–nutrient interactions exacerbate sensitivity to warming in phytoplankton. Global Change Biology. 2017;23: 3269–3280. doi:10.1111/gcb.13641
