# Supplementary material for: The effects of temperature and pH on the reproductive ecology of sand dollars and sea urchins: Impacts on sperm swimming and fertilization
Source: PLoS One. 2022 Dec 1;17(12):e0276134. doi: 10.1371/journal.pone.0276134 (PMC9714736; doi:10.1371/journal.pone.0276134)
Supplement: S3 Table — Models ranked by small-sample corrected Akaike Information Criterion (AICc) and Bayesian Information Criterion (BIC) values for each reproductive trait. (DOCX) [file pone.0276134.s005.docx]

**Table S3.** Fitted thermal performance curve models ranked by small-sample corrected Akaike Information Criterion (AICc) and Bayesian Information Criterion (BIC) values for each reproductive trait.

**Sand dollars**

|  | Fertilization | | Sperm velocity | | Motility | |
| --- | --- | --- | --- | --- | --- | --- |
| Model | AICc | BIC | AICc | BIC | AICc | BIC |
| Beta | 270.034 | 276.64 | 352.585 | 359.189 | 302.957 | 309.56 |
| Boatman | 302.656 | 276.90 | 352.178 | 358.783 | 306.182 | 309.57 |
| Delong | 282.876 | 289.481 | 354.713 | 361.318 | 304.321 | 310.925 |
| Flinn | 290.913 | 295.957 | 351.356 | 356.400 | 301.254 | 306.298 |
| Gaussian | 275.346 | 280.39 | 376.147 | 381.19 | **298.730** | **303.77** |
| Jöhnk | 270.316 | 276.83 | 376.650 | 383.255 | 303.066 | 309.67 |
| Modified Gaussian | 269.961 | 275.88 | **348.419** | **354.337** | 301.422 | 307.34 |
| Quadratic | **264.640** | **269.68** | 368.184 | 373.227 | 300.681 | 305.72 |
| Thomas | 278.239 | 284.844 | 395.139 | 401.744 | 306.552 | 313.156 |
| Weibull | 270.544 | 276.46 | 376.172 | 382.09 | 301.336 | 307.25 |

**Red urchins**

|  | Fertilization | | Sperm velocity | | Motility | |
| --- | --- | --- | --- | --- | --- | --- |
| Model | AICc | BIC | AICc | BIC | AICc | BIC |
| Beta | 167.6875 | 167.9546 | 191.3445 | 191.6116 | 186.2934 | 186.5606 |
| Boatman | 167.6428 | 167.91 | 191.1993 | 191.4664 | 186.7102 | 186.9774 |
| Delong | 179.7519 | 180.019 | 195.1053 | 195.3724 | 187.2548 | 187.522 |
| Flinn | 181.0301 | 182.7082 | 190.2378 | 191.9159 | 181.4267 | 183.1048 |
| Gaussian | 172.1612 | 173.8393 | 188.3383 | 190.0164 | 179.5745 | 181.2526 |
| Jöhnk | 169.6167 | 169.8839 | 191.986 | 192.2532 | 186.0834 | 186.3505 |
| Modified Gaussian | **162.8711** | **164.0938** | 188.8216 | 190.0442 | 181.7831 | 183.0057 |
| Quadratic | 166.8118 | 168.4899 | **186.736** | **188.4141** | **178.9923** | **180.6704** |
| Thomas | 188.3085 | 188.5756 | 204.2043 | 204.4715 | 186.6068 | 186.874 |
| Weibull | 174.1868 | 175.4094 | 187.807 | 189.0296 | 182.3178 | 183.5404 |
