# Supplementary material for: The effects of temperature and pH on the reproductive ecology of sand dollars and sea urchins: Impacts on sperm swimming and fertilization
Source: PLoS One. 2022 Dec 1;17(12):e0276134. doi: 10.1371/journal.pone.0276134 (PMC9714736; doi:10.1371/journal.pone.0276134)
Supplement: S4 Table — Parameters that differ significantly between acclimation treatments are shown in bold. (DOCX) [file pone.0276134.s006.docx]

**Table S4.** Parameter estimates with 95% confidence intervals from thermal performance curves of sand dollars and sea urchins. T_opt_ = optimum temperature (◦C); T_br_ = thermal breadth (◦C) and; r_max_ = maximum rate. Parameters that differ significantly between acclimation treatments are shown in bold.

| Variable | Parameter | Sand dollars | Red urchins |
| --- | --- | --- | --- |
| Fertilization rate | t_opt_ | 17.571 (15.690, 18.610) | 16.661 (15.704, 17.215) |
|  | **T_br_** | 18.327 (17.638, 19.5) | 16.042 (15.461,16.052) |
|  | r_max_ | 91.265 (87.918, 94.409) | 84.033 (79.967, 88.489) |
| Sperm velocity | **t_opt_** | 13.881 (12.326,14.706) | 19.41 (18.22, 20.86) |
|  | **T_br_** | 9.664 (7.196,12.258) | 14.176 (12.6197, 16.647) |
|  | **r_max_** | 274.015 (246.347, 293.837) | 160.663 (149.699,174.919) |
| Sperm motility | t_opt_ | 13.082 (9.118,14.742) | 10.86 (8,14.527) |
|  | T_br_ | 12.521 (10.275, 14.570) | 11.886 (6.374,13.739) |
|  | r_max_ | 72.489 (66.278, 79.093) | 80.25 (71.77, 86.79) |
